# Quantitative elementary mode analysis of metabolic pathways: the example of yeast glycolysis

**DOI:** 10.1186/1471-2105-7-186

**Published:** 2006-04-03

**Authors:** Jean-Marc Schwartz, Minoru Kanehisa

**Affiliations:** 1 Bioinformatics Center, Institute for Chemical Research, Kyoto University, Uji, Kyoto 611-0011, Japan

## Abstract

**Background:**

Elementary mode analysis of metabolic pathways has proven to be a valuable tool for assessing the properties and functions of biochemical systems. However, little comprehension of how individual elementary modes are used in real cellular states has been achieved so far. A quantitative measure of fluxes carried by individual elementary modes is of great help to identify dominant metabolic processes, and to understand how these processes are redistributed in biological cells in response to changes in environmental conditions, enzyme kinetics, or chemical concentrations.

**Results:**

Selecting a valid decomposition of a flux distribution onto a set of elementary modes is not straightforward, since there is usually an infinite number of possible such decompositions. We first show that two recently introduced decompositions are very closely related and assign the same fluxes to reversible elementary modes. Then, we show how such decompositions can be used in combination with kinetic modelling to assess the effects of changes in enzyme kinetics on the usage of individual metabolic routes, and to analyse the range of attainable states in a metabolic system. This approach is illustrated by the example of yeast glycolysis. Our results indicate that only a small subset of the space of stoichiometrically feasible steady states is actually reached by the glycolysis system, even when large variation intervals are allowed for all kinetic parameters of the model. Among eight possible elementary modes, the standard glycolytic route remains dominant in all cases, and only one other elementary mode is able to gain significant flux values in steady state.

**Conclusion:**

These results indicate that a combination of structural and kinetic modelling significantly constrains the range of possible behaviours of a metabolic system. All elementary modes are not equal contributors to physiological cellular states, and this approach may open a direction toward a broader identification of physiologically relevant elementary modes among the very large number of stoichiometrically possible modes.

## Background

### Network-based pathway analysis

Biological research in the twentieth century has been dominated by the reductionist approach, providing valuable information about the properties and functions of individual cellular components. But the behaviour of complex systems of interacting components cannot be comprehended by the sole characterisation of their individual components or pair-wise relations, because new properties emerge from the interaction of large numbers of components. Technological developments now allow to gain more and more knowledge about the various interaction networks that govern cellular properties, including protein-protein interactions, metabolic pathways, regulatory and signalling networks. Therefore systems-based approaches are now needed with the aim of modelling and understanding the complex cellular processes producing biological functions, and in the long term providing an integrated and predictive description of a complete cell [1].

Metabolic pathways are an essential key to the systemic behaviour of a biological cell. Two types of approaches are generally possible for the study of cellular metabolism. The first type involves detailed modelling of the dynamical features of biochemical networks. Several tools have been created for simulating metabolic and signalling networks in biological cells [2-5]. However the accurate experimental determination of kinetic functions and parameters is a difficult and time-consuming task, that can only be thoroughly performed for small networks of particular interest. In contrast, a second type of approaches have been developed that use only the topological and stoichiometric properties of metabolic networks, which are usually well known. This information is sufficient to determine a set of constraints limiting the range of possible states of a metabolic system in steady-state. With flux-balance analysis [6], an optimal solution can then be found in the space of possible behaviours by maximising a function of interest, for example growth rate, using linear optimisation. These approaches have become useful tools for analysing and assessing the capabilities of metabolic networks [7,8].

In such network-based pathway analyses, metabolic networks are represented by stoichiometric matrices that relate reactions and metabolites. These matrices are analysed by algorithms that compute particular sets of routes satisfying specified conditions. Two very similar concepts called *elementary modes *and *extreme pathways *have been introduced in recent years [9-11]. An elementary mode is a minimal set of reactions that can operate in steady state, while the set of extreme pathways is the systemically independent subset of the elementary modes. In a mathematical multidimensional representation where each axis corresponds to a reaction flux, all possible steady-state flux distributions lie within a multidimensional flux cone. Extreme pathways form the edges of this cone, and the additional elementary modes may lie on the surface or in the interior of the cone.

Applications of network-based pathway analyses have been presented for predicting functional properties of metabolic networks, measuring different aspects of robustness and flexibility, and even assessing gene regulatory features [12,13]. [14] showed that a combination of metabolome and elementary mode analysis on a stoichiometric model of *Saccharomyces cerevisiae *provided a framework for the identification of functions of orphan genes. [15] presented an extreme pathway analysis of amino acid producing pathways in *Haemophilus influenzae*, showing the significance of this approach to understanding the functional properties of metabolic networks at the genome scale. Elementary modes can provide a measure of structural robustness of metabolic networks, as has been shown by a comparative study of the central metabolisms of *Escherichia coli *and human erythrocytes [16]. Other examples of elementary mode or extreme pathway based applications include the study of the mechanisms of sucrose accumulation in sugar cane [17], the physiological interpretation of red blood cell metabolism [18], the investigation of photosynthate metabolism in the chloroplast strauma [19], and the analysis of the central carbon metabolism of *Saccharomyces cerevisiae *[20].

The two concepts of elementary modes and extreme pathways are very similar but not identical, the crucial difference being that extreme pathways are defined as systemically independent, which means that no extreme pathway can be represented as a non-negative linear combination of other extreme pathways. Comparisons between both concepts and discussions about their advantages and drawbacks have been published [21-23]. If only irreversible exchange reactions are present in a system, the two sets are equivalent. Otherwise, extreme pathways are fewer in number, which can be of advantage for the analysis of large systems. However, extreme pathways may have to be added together to represent a particular flux state that cancels out a reversible exchange flux, and such occurrences complicate biological interpretation. For this reason, we chose to base this work on elementary modes, although a similar approach could be undertaken on the basis of extreme pathways.

### Decomposition of steady-state flux distributions

Although the space generated by elementary modes or extreme pathways contains all possible steady-state flux distributions, not necessarily all these states may actually be reached by a real biological organism. Several approaches have already been proposed in order to further characterise the space of allowable states. Regulatory constraints repressing gene expression have been modelled, and it has been shown that they strongly reduce the number of allowed extreme pathways in specific environmental conditions [24]. Singular value decomposition of stoichiometric matrices has lead to the identification of dominant modes that correspond to relevant physiological metabolic states [25].

A first attempt to understand how physiological steady states can be reconstructed from a network's extreme pathways lead to the concept of *α-spectrum *[26]. Any flux distribution in a metabolic network can be described as a linear combination of elementary modes or extreme pathways, but this decomposition is generally not unique. The *α*-spectrum describes the range of possible weightings a particular mode can take in the decomposition. This range is determined using linear optimisation to maximise and minimise the weighting of a particular extreme pathway in the reconstruction. A drawback of that description is that the range of allowable weights for a given extreme pathway is not necessarily independent of the weight value taken by any other extreme pathway.

In a previous communication, we presented a different decomposition approach that assigns each elementary mode a unique weight [27,28]. Because the set of possible decompositions is usually a continuous convex space of non-zero dimension, an additional constraint had to be introduced. We proposed to select the particular set that minimises the length of the weight vector, because this decomposition makes maximum use of the modes that are closest to the actual state of the system and are therefore most relevant for biological interpretation. Starting from a different approach, [29] introduced a flux decomposition method that lead to very similar results. Therefore, the objectives of this article are twofold: first, to provide a detailed description of our algorithm allowing a comparison of both approaches (see Methods section); second, to show how such decompositions can be used in combination with kinetic modelling to provide a framework for the characterisation of elementary mode usage in real metabolic systems, and for assessing the effects of changes in enzyme kinetics on the distribution of metabolic processes.

## Results

### Model

Our approach is illustrated using a model of yeast glycolysis presented by [30]. This model is available from the JWS online repository [31]. It was constructed after experimental determination of all kinetic parameters, and can therefore be assumed to represent physiologically accurate metabolic states. In [30] an unbranched model was first developed, but was unable to reach a steady state when experimentally determined parameter values were introduced. Therefore glycogen, trehalose, glycerol, and succinate branches were added. No precise kinetic modelling was applied to the glycogen and trehalose branches, but fluxes were simply assigned constant values corresponding to experimental measurements. All flux values in the following sections (including elementary mode fluxes) are expressed in mmol·min^-1^·l^-1^, and concentrations are expressed in mmol·l^-1^. Abbreviations used for enzyme and compound names are listed in Table 1.

**Figure 1:**
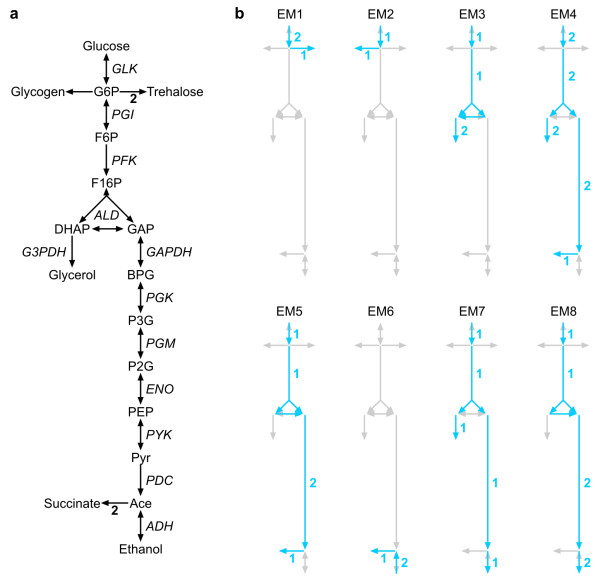
**Model of yeast glycolysis**. a. Model of yeast glycolysis introduced by [30]. Names in italic are enzymes whose kinetic properties were included in this analysis. Numbers indicate stoichiometries of reactions when different from one. b. The eight elementary modes of the yeast glycolysis system. All elementary modes are irreversible. External metabolites are: glucose, glycogen, trehalose, glycerol, succinate, and ethanol.

The system and its eight elementary modes are shown in Figure 1. For elementary modes to be calculated, external metabolites representing the entry and exit points of a system must be defined. In the present case their definition is straightforward: glucose, glycogen, trehalose, glycerol, succinate, and ethanol are considered to be external metabolites. All elementary modes of this system are irreversible, since they all contain at least one irreversible reaction. Therefore no negative elementary mode flux is allowed for any of them.

As shown earlier [28], decomposition of the experimental steady state assigned the highest flux to EM8 (elementary mode flux value of 55.5), which is the standard glycolytic path leading to the production of ethanol from glucose. The second highest flux (18.2) was assigned to EM7, which combines the production of ethanol with the derived production of glycerol from DHAP. All other modes were assigned low or zero fluxes.

It should be noted that the cases of EM1 and EM2 are particular. These elementary modes depend entirely on the trehalose and glycogen branches respectively. These two branches retain constant fluxes in the model, therefore the fluxes carried by EM1 and EM2 always remain constant as well. As a result, these modes cannot be further analysed and will not be further discussed. Nevertheless, we chose to keep them in our analysis to maintain consistency with the model from [30], and because the glycogen and trehalose branches have been mentioned to help the system in reaching a steady state.

### Influence of individual kinetic parameters

In this section, we studied variations in elementary mode fluxes induced by variations of individual kinetic parameters, all other parameters of the model being kept at their reference values. This case may apply to the situation where a given parameter has not been experimentally measured or a large uncertainty exists for its value. For example, very different values of the *L*_0 _parameter in the PFK kinetic relation can be found in the literature: a value of 3342 was used in [32], while 0.66 was used in [30].

The Gepasi software [33] was used to compute steady states for a range of values of each parameter, and all steady-state flux distributions were decomposed onto elementary modes using our algorithm. The bounds of intervals of parameter values were chosen as the approximate extreme values for which the Gepasi software was able to compute a steady-state flux distribution. Those bounds are listed in Table 2 for all parameters, together with their reference values given in [30].

The obtained standard deviations of the distributions of elementary mode fluxes are given in Table 3. EM1 and EM2 show no variation for the reason mentioned earlier. EM6 shows no variation either, although flux in the succinate branch is not constant; the two other modes contributing to that flux (EM4 and EM5) indeed do show variations.

**Figure 2:**
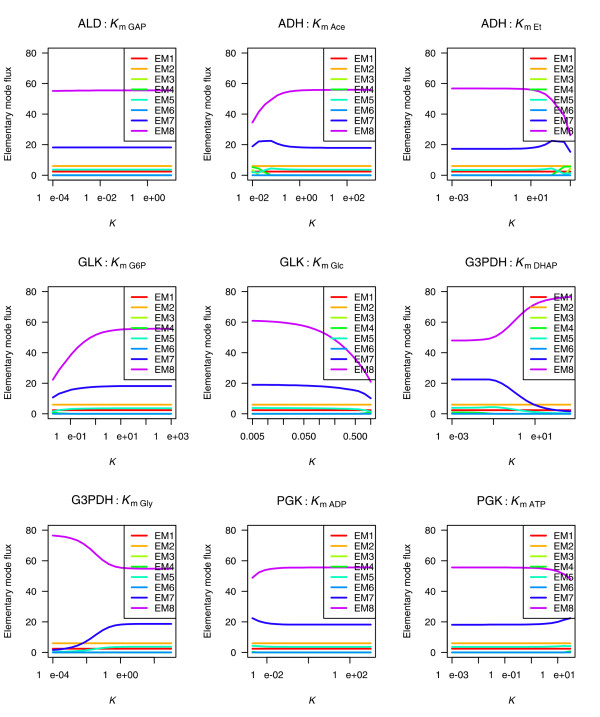
**Effects of kinetic parameters on elementary mode fluxes**. Effects of single kinetic parameters on elementary mode fluxes. In each example, the variable parameter is indicated at the top of the graph, and all other parameters were kept at their reference values given in Table 2. Horizontal axes use logarithmic scales.

In most cases, the effect of variation of a single kinetic parameter on the distribution of elementary mode fluxes is weak. Several parameters have almost no effect: the most striking examples are the parameters of ALD (Figure 2) and PDC. Most parameters of PFK have limited influence too, although important efforts have been invested into modelling the kinetics of this enzyme.

Effects of some parameters are characterised by a large range of stability, where parameter variations have very little effect on elementary mode fluxes, followed or preceded by a decrease of most fluxes when approaching the limits of the steady-state domain. This type of behaviour is produced for example by *K*_m·Ace _and *K*_m·Et _of ADH, *K*_m·G6P _and *K*_m·Glc _of GLK (Figure 2). It can be noted that a decrease in the dominant elementary modes EM8 and EM7 is sometimes accompanied by an increase in EM3 and EM4, indicating that the system tends to shift toward higher glycerol and succinate producing states in those areas. However these modes are never able to gain high values, as the system soon leaves the steady-state domain.

For a third type of parameters, a range of stability followed or preceded by variations can be observed as well, but here the variation of EM8 is accompanied by the opposite variation of EM7 and, to a smaller extend, of EM5 and EM4. In the case of *K*_m·DHAP _and *K*_m·Gly _of G3PDH (Figure 2), a second stable area seems to be reached, characterised by a very low flux for EM7 and the almost exclusive use of EM8. In the case of *K*_m·ADP _and *K*_m·ATP _of PGK on the opposite, EM7 seems to tend toward higher values, but the limits of the steady-state domain are rapidly reached.

### Influence of individual enzymes

In this section, all the parameters of a given enzyme kinetic relation were allowed to vary independently from each other. For each enzyme, 400 sets of parameter values were chosen randomly in the ranges listed in Table 2. Table 4 shows the standard deviations of the distributions of elementary mode fluxes obtained for every enzyme. The trends observed in Table 2 are mostly confirmed. The enzymes whose kinetics have the largest influence on the system are G3PDH, GAPDH and ADH, while ALD on the opposite has almost no effect.

**Figure 3:**
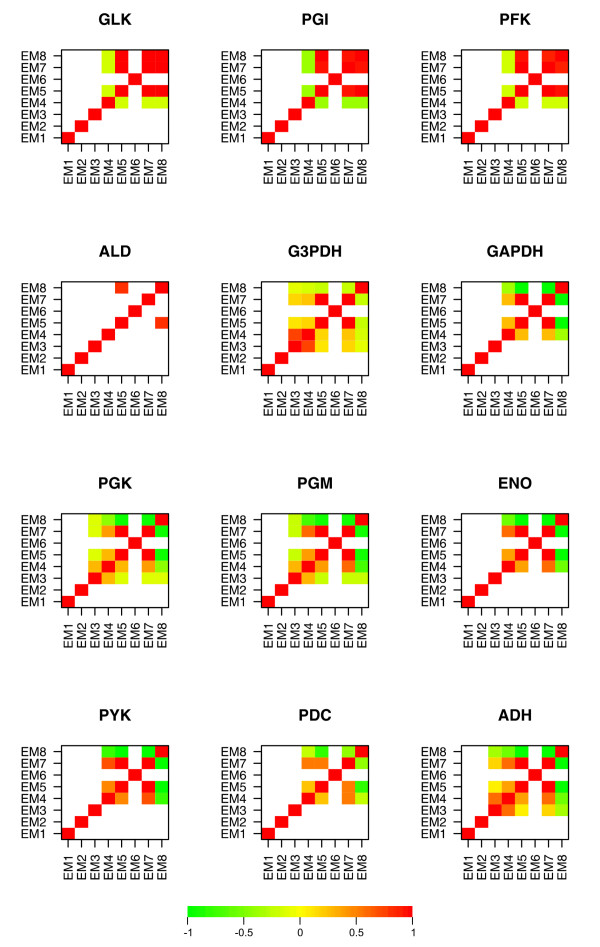
**Correlations between elementary mode fluxes induced by individual enzymes**. Correlations between variations of pairs of elementary mode fluxes under random variations of all kinetic parameters of each enzyme. Values are colour-coded according to the bar at the bottom of the figure. White squares indicate that no correlation coefficient could be calculated, *i.e. *that one of the two elementary modes retained a constant flux value.

The correlation coefficients between pairs of elementary mode fluxes are shown in Figure 3. According to these maps, the effects of enzymes in the glycolytic system can be grouped into several categories:

- ALD kinetics has little effect on the system. The only observed variations are for EM7 and EM8, and they are strongly correlated. This indicates that ALD may only influence the system globally, but not the balance of its different branches.

- GLK, PGI and PFK produce similar effects. The responses of EM5, EM7 and EM8 are strongly correlated for these enzymes. This may be explained by the fact that these enzymes are located in the top branch of the system, and are thus unable to affect the balance of branches located further down. However the response of EM4 is not correlated to the three other modes. EM4 actually involves two divergences from the dominant mode EM8, whereas EM7 and EM5 involve only one. Therefore several independent processes may lead to an activation of EM4, explaining its decorrelation from EM8.

- G3PDH produces strong correlations between EM5 and EM7, and between EM3 and EM4, but these groups are only weakly correlated with each other, and not at all with EM8. G3PDH controls the glycerol branch, and it looks as if flux changes in the glycerol branch are compensated by changes in other side branches independently of the dominant regime EM8.

- GAPDH, PGK, PGM, ENO, and PYK exhibit related effects as well, whose most striking characteristics are an anti-correlation of EM8 with EM5 and EM7. When kinetic changes in these enzymes result in an alteration of the production of ethanol, they are compensated by the production of glycerol and succinate. EM4 is also correlated with EM5 and EM7, but EM3, when effects can be noticed, is not. Correlations are not as clear for PDC, although this enzyme would be expected to produce similar effects because of its localisation in the same branch as that group of enzymes. PDC kinetics is actually modelled by a simple two-compound Michaelis-Menten relation, and a more advanced model may be required.

- ADH exhibits similar correlations as the previous group. The most notable effects of ADH kinetics would have been expected on EM6, but because this mode was not activated in any calculated steady state, no such effects could be observed.

### Influence of external concentrations

**Figure 4:**
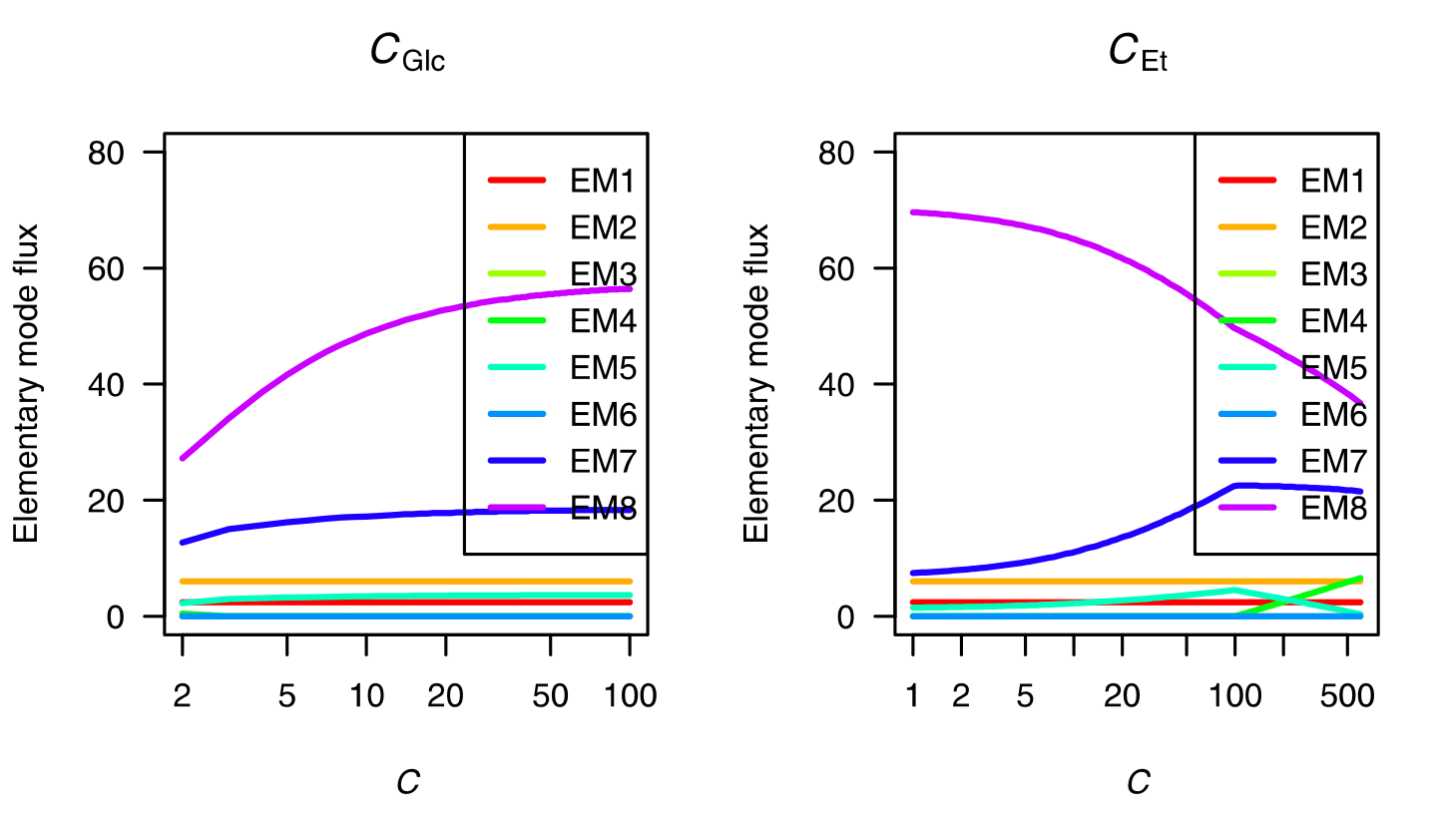
**Effects of external concentrations on elementary mode fluxes**. Effects of external concentrations of glucose (left) and ethanol (right) on elementary mode fluxes. In both cases, the non-variable concentration was 50, and all kinetic parameters were kept at their reference values given in Table 2. Horizontal axes use logarithmic scales.

We additionally looked at the effects of variations in concentrations of external compounds on elementary mode fluxes. Only two external concentrations are defined in the glycolysis model: glucose and ethanol. The effect of a variation of glucose concentration is not very different from what was observed for some kinetic parameters, such as *K*_m·G6P _of GLK (Table 5 and Figure 4). For low glucose concentrations, all fluxes tend to decrease but the balance of the different modes is not significantly affected. Changes in ethanol concentration produce more complex effects. For very high concentrations of external ethanol, the production of ethanol from acetaldehyde is slowed down and the system shifts toward glycerol and succinate production. But even under these conditions, no contribution could be found for EM6, as the system never reached a state where the ADH reaction would work in the reverse direction (*i.e. *production of acetaldehyde from ethanol).

**Figure 5:**
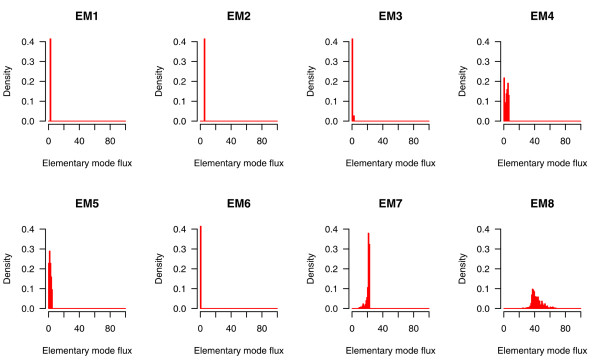
**Elementary mode fluxes spanned by external concentrations**. Distributions of elementary mode fluxes obtained for 400 random sets of glucose and ethanol concentration values. Glucose concentration was allowed to vary between 2 and 100, ethanol concentration between 1 and 600. Vertical axes have been cut at 0.4 but the height of peaks for constant modes extends till 1.

The distributions of elementary mode fluxes obtained for 400 random glucose and ethanol concentration values are shown in Figure 5, and confirm the trends revealed by Figure 4. Most elementary modes have a very narrow distribution around their reference value, while only EM8 has a broader distribution ranging from approximately 30 to 70.

### Exploring the space of attainable steady states

We subsequently attempted to span the entire space of kinetically attainable steady states by allowing all kinetic parameters of all enzymes to vary simultaneously in the ranges defined in Table 2. Because the dimension of the parameter space is too large for it to be scanned is a systematic way, parameter values were selected randomly in their allowed intervals. 400 random sets of parameters were selected, and for each of them the steady-state flux distribution was computed using the Gepasi software and decomposed onto elementary modes using our algorithm.

**Figure 6:**
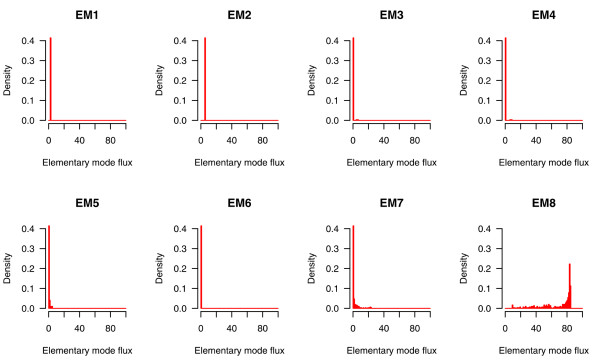
**Elementary mode fluxes spanned by kinetic parameters**. Distributions of elementary mode fluxes obtained for 400 random sets of values of all kinetic parameters in the system. Vertical axes have been cut at 0.4 but the height of peaks for constant modes extends till 1.

Figure 6 shows the histograms of the distributions obtained for the eight elementary mode fluxes. The fluxes of EM1 and EM2 always remained constant for the reason stated earlier. The flux of EM6 always remained at zero, although this condition is not dictated by the kinetics of the succinate and ADH reactions. The only possibility for EM6 to achieve a non-zero flux would be for the ADH reaction to consume ethanol and produce acetaldehyde, and such a state was never reached by this model. A change in the kinetic representation of this system may therefore be required if it is to model the shift from glycolysis to respiration. The fact that EM3 remained little used indicates that the triose-phosphate isomerase reaction is driven in most cases to the direction of GAP.

The distributions of EM3, EM4, EM5 and EM7 all show very little variation and are concentrated at low values. Only EM8 shows a broader distribution spanning most of the possible flux values, with an important peak around 80. A majority of steady states thus represent distributions where most of the flux in the system is consumed by EM8, with very little use of the other elementary modes. Interestingly, this dominant state is not identical to the experimental steady state, where EM8 is still the most important mode but EM7 consumes a non-negligible proportion of flux.

**Figure 7:**
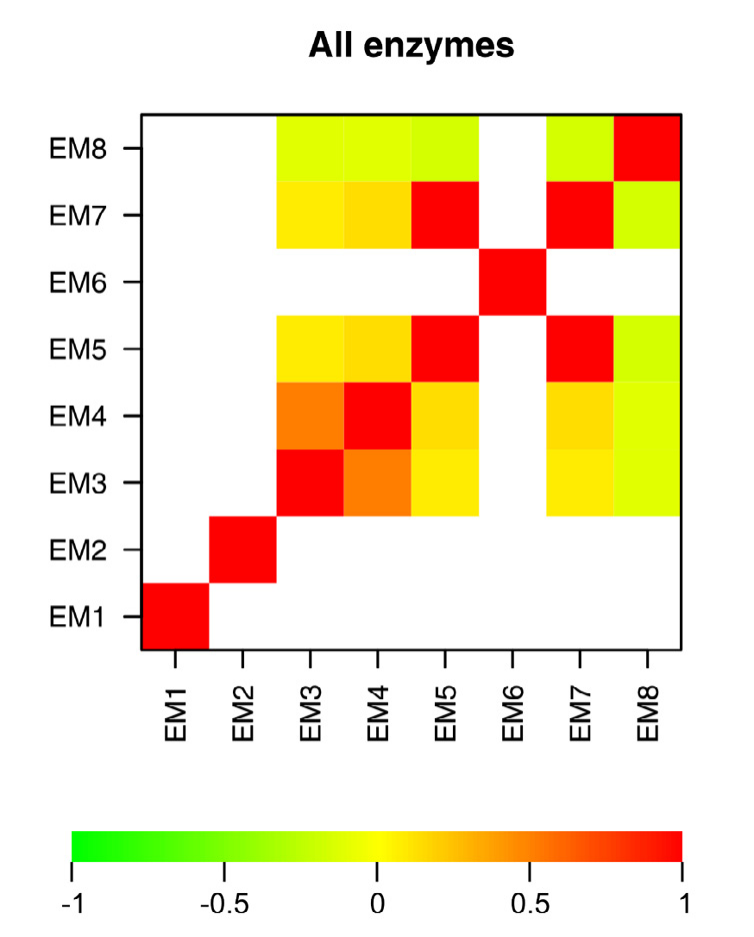
**General correlations between elementary mode fluxes**. Correlations between variations of pairs of elementary mode fluxes under random variation of all kinetic parameters of all enzymes. Values are colour-coded according to the bar at the bottom of the figure. White squares indicate that no correlation coefficient could be calculated, *i.e. *that one of the two elementary modes retained a constant flux value.

Correlations between the different elementary mode fluxes were computed as in the previous section (Figure 7). EM5 and EM7, respectively EM3 and EM4, show strong positive correlations. This observation is consistent with the results obtained for individual enzymes, as the correlations between those pairs were positive in most cases (Figure 3). The remaining pairs of elementary modes are not correlated, which is also consistent with the previous observations. In the relation between EM7 and EM8 for example, GLK, PGI, and PFK created a positive correlation, while GAPDH, PGK, PGM, ENO, PYK, PDC, and ADH created a negative correlation.

### Relations to metabolic control analysis

The approach followed in the previous sections presents resemblances to methods developed in the framework of Metabolic Control Analysis (MCA) [34]. The major difference between both approaches is that MCA is based on the analysis of small parameter variations around a given state, while our approach on the contrary was aimed at spanning an as large as possible range of possible states. Unless MCA were repeated for a large number of different states (which would be prohibitive given the large number of parameters), it does not allow a description of the distributions and correlations between elementary mode fluxes in large intervals.

In order to clarify the relations between both approaches, we nevertheless applied MCA to the most significant state for this system: the experimental steady state determined by [30]. Two types of coefficients have been defined in MCA. First, elasticity coefficients describe the effects of individual parameters on the rate of the given reaction considered to be isolated:

εpv=∂v∂ppv     (1)
 MathType@MTEF@5@5@+=feaafiart1ev1aaatCvAUfKttLearuWrP9MDH5MBPbIqV92AaeXatLxBI9gBaebbnrfifHhDYfgasaacH8akY=wiFfYdH8Gipec8Eeeu0xXdbba9frFj0=OqFfea0dXdd9vqai=hGuQ8kuc9pgc9s8qqaq=dirpe0xb9q8qiLsFr0=vr0=vr0dc8meaabaqaciaacaGaaeqabaqabeGadaaakeaaiiGacqWF1oqzdaqhaaWcbaGaemiCaahabaGaemODayhaaOGaeyypa0ZaaSaaaeaacqGHciITcqWG2bGDaeaacqGHciITcqWGWbaCaaWaaSaaaeaacqWGWbaCaeaacqWG2bGDaaGaaCzcaiaaxMaadaqadaqaaiabigdaXaGaayjkaiaawMcaaaaa@3EDA@

where *p *is the perturbation parameter and *v *the rate for the isolated enzyme. Second, control coefficients describe the effects of a given enzyme on system fluxes, by comparing a rate variation induced by a perturbation for the isolated enzyme to the variation of system flux induced by the same perturbation. Control coefficients can be defined in a similar way for elementary mode fluxes:

Cvw=δwδvvw     (2)
 MathType@MTEF@5@5@+=feaafiart1ev1aaatCvAUfKttLearuWrP9MDH5MBPbIqV92AaeXatLxBI9gBaebbnrfifHhDYfgasaacH8akY=wiFfYdH8Gipec8Eeeu0xXdbba9frFj0=OqFfea0dXdd9vqai=hGuQ8kuc9pgc9s8qqaq=dirpe0xb9q8qiLsFr0=vr0=vr0dc8meaabaqaciaacaGaaeqabaqabeGadaaakeaacqWGdbWqdaqhaaWcbaGaemODayhabaGaem4DaChaaOGaeyypa0ZaaSaaaeaaiiGacqWF0oazcqWG3bWDaeaacqWF0oazcqWG2bGDaaWaaSaaaeaacqWG2bGDaeaacqWG3bWDaaGaaCzcaiaaxMaadaqadaqaaiabikdaYaGaayjkaiaawMcaaaaa@3EE7@

where *v *is the rate of the isolated enzyme and *w *is an elementary mode flux in the system. It should be noted that control coefficients of a given reaction are independent on the choice of the perturbation parameter, therefore only one control coefficient exists characterising the effect of a given enzyme onto a given systemic flux [35]. Elasticity coefficients on the contrary are local properties that are not linked to a particular system.

Elasticity coefficients of parameters of the glycolysis model are shown in the last column of Table 3, and control coefficients are shown in Table 6. No relation can be observed between standard deviations of elementary mode fluxes over large intervals and elasticity coefficients. For example *V*_max _and *K*_eq _of G3PDH have very different elasticities, while their effects on elementary mode fluxes are in the same range. As was shown in Figure 2, some parameters have very different effects in different areas, and local parameters are not sufficient for characterising effects over wide intervals.

Control coefficients cannot be calculated at the experimental steady state for EM3, EM4, and EM6, because both *δw *and *w *are equal to zero for these modes. For the remaining elementary modes, standard deviations over large intervals cannot be inferred from local control coefficients in general. For example, GLK and PGI produce similar large-range effects but have very different local control coefficients on EM8. On the opposite, G3PDH and GAPDH have both strong large-range effects and high local control coefficients on several modes. The overall correlation between EM5 and EM7 can be observed locally, as values of all control coefficients are very similar for these two modes. In order to check that the summation relationship of flux control coefficients also applies to elementary mode fluxes, we added to Table 6 the processes corresponding to glucose transport, conservation of adenine nucleotides, and branches with approximate modelling in [30]. The sums of control coefficients of elementary mode fluxes are indeed close to unity, apart from computational approximations.

## Discussion

The motivation for providing a decomposition of flux distributions onto elementary modes was to provide a quantitative measure of the utilisation of each elementary mode in a metabolic system under given conditions. Each elementary mode represents a particular route of transformation of some substrates into some products, and can therefore be viewed as an elementary possible biological function of a metabolic pathway. Having a measure of elementary mode utilisation is important for two reasons. First, it makes it possible to observe which elementary modes are significantly active in a biological system and which ones are not. This capability greatly enhances the biological interpretability of elementary mode based pathway analyses, and allows to concentrate further investigations on the physiologically active processes among the usually very large number of stoichiometrically possible processes. Second, a quantitative measure makes it possible to quantify the effects of changes in a system (e.g. in the concentration of some metabolite, in the kinetics of some reaction, or in genetic expression leading to the induction or repression of some reaction) on the redistribution of metabolic processes in that system. Such analyses may in turn be used in a biotechnology perspective for identifying components which have the strongest effect on some desirable physiological process. In our example, several kinetic parameters were shown to have very little effect on the steady state of the glycolytic system, while a smaller set of parameters can account for significant variations. The same approach may be relevant when attempting to model particular biochemical reactions based on experimental measurement of kinetic parameters, as the most important parameters could be identified in order to concentrate experimental efforts on them.

It is particularly interesting that the same choice for a relevant decomposition, *i.e. *minimising the norm of the elementary mode flux vector, was introduced independently by two different teams based on two different approaches. We previously justified this choice from a geometrical and biological point of view [27,28]. Our approach was based on the idea that the *best *decomposition should assign maximum weights to the modes that are closest to the actual state of the system, *i.e. *the modes that best describe it biologically. The authors of [29] in turn showed that the same choice results from using the Moore-Penrose generalised inverse for inverting the matrix of elementary modes. They furthermore showed that this decomposition possesses desirable mathematical properties such as accuracy, nullity, computability, and continuity in the case where all reactions are reversible. Continuity cannot be guaranteed yet when irreversible reactions are present, although no single occurrence of discontinuity was observed in all our computations.

In the glycolytic system, our analysis found the space of kinetically feasible steady states to be significantly smaller than the space of stoichiometrically feasible steady states. When only stoichiometry is taken into account, all linear combinations of reversible elementary modes and non-negative linear combinations of irreversible elementary modes are valid steady states. But when a kinetic model is applied, it appears that a significantly smaller part of the steady state space is actually attainable, even when large ranges of values are allowed for all kinetic parameters. Most elementary mode fluxes in the glycolytic system were constrained to narrow intervals, and only two elementary mode fluxes could span large intervals of values. These results are consistent with other findings on cellular metabolism. A system-wide analysis of metabolic fluxes in *Escherichia coli *based on linear programming revealed that the overall activity of metabolism is dominated by a high-flux backbone, while most other reactions have low fluxes [36]. This reaction core appeared to be robust to environmental perturbations and evolutionary conserved [37]. A number of experimental analyses also revealed that most flux control coefficients of metabolic pathways are small [38], indicating that perturbations in most enzymes have little effect on metabolic fluxes. This robustness may be a inherent property of enzyme systems, since the opposite would have deleterious effects on cell metabolism. It therefore appears reasonable to expect the same properties for elementary mode fluxes. We would hypothesize that only a small number of elementary modes have significant activity in large systems as well, and that most elementary mode fluxes should be little affected by changes in enzyme kinetics. The approach presented here may open a direction toward identifying, among the large number of stoichiometrically possible elementary modes, a smaller set of physiologically significant elementary modes that are suitable to biological interpretation.

A genome-wide identification of such modes by the approach presented here remains a long process though, and further efforts should be directed toward developing automatic procedures combining such kinetic and elementary mode analyses. The decomposition algorithm itself is fast and converges in a fraction of a second for the glycolysis example. Quadratic programming problems defined by positive-definite Hessian matrices (as is the case here) can be solved in polynomial time [39], but performance will significantly decrease in large systems. However, possibilities exist for reducing the dimensions of systems to be decomposed. Enzymes that operate together in fixed flux proportions in all steady states can be grouped and reduced to a single reaction, as was presented by [40]. In addition, procedures have been presented for decomposing large metabolic networks into subnetworks, and it was shown that the computation of elementary modes itself is more efficient when applied to several small systems than one large system [41]. The same procedures can be followed to obtain systems that will be sufficiently small for flux decompositions to be performed efficiently.

## Conclusion

We showed how a combination of kinetic modelling and elementary mode based flux decompositions makes it possible to analyse the distribution of metabolic processes in physiological cellular states, and to assess the effects of kinetic changes onto the balance of utilisation of different elementary modes. Similar approaches as the one presented here could be applied to understand the metabolic response to external perturbations or genetic changes, to identify possible genetic alterations for the optimisation of metabolic processes of particular interest, or to quantitatively measure and compare the effect of different drugs on cellular functions. Elementary modes indeed represent elementary possible metabolic functions, and the set of elementary modes provides a basis for the functional interpretation of biochemical systems. We believe that further efforts should be directed toward fully exploiting this potential, including the systematic computation and functional annotation of elementary modes in complete metabolic systems.

## Methods

### Decomposition of flux distributions

We consider a metabolic network with *m *elementary modes and *n *reactions. The elementary modes are represented by vectors **E**_1_, **E**_2_...**E**_*m*_, each of them being of length *n*. A flux distribution in this system is represented by a vector **v **of length *n*. A decomposition of this flux distribution onto the set of elementary modes is defined by a vector **w **of length *m *such that:

v=∑j=1mwjEj     (3)
 MathType@MTEF@5@5@+=feaafiart1ev1aaatCvAUfKttLearuWrP9MDH5MBPbIqV92AaeXatLxBI9gBaebbnrfifHhDYfgasaacH8akY=wiFfYdH8Gipec8Eeeu0xXdbba9frFj0=OqFfea0dXdd9vqai=hGuQ8kuc9pgc9s8qqaq=dirpe0xb9q8qiLsFr0=vr0=vr0dc8meaabaqaciaacaGaaeqabaqabeGadaaakeaaieqacqWF2bGDcqGH9aqpdaaeWbqaaiabdEha3naaBaaaleaacqWGQbGAaeqaaOGae8xrau0aaSbaaSqaaiabdQgaQbqabaaabaGaemOAaOMaeyypa0JaeGymaedabaGaemyBa0ganiabggHiLdGccaWLjaGaaCzcamaabmaabaGaeG4mamdacaGLOaGaayzkaaaaaa@3F87@

Given that the elements of **E**_*j *_are dimensionless, those of **w **are expressed in units of flux. We previously used to refer to *w*_*j *_values as *elementary mode weightings *[28], but in order to highlight the fact that they indeed represent fluxes carried by individual elementary modes and to establish consistency with [29], we refer to them from now as *elementary mode fluxes*. This terminology furthermore highlights the difference between *w*_*j *_values and *α-weightings *as introduced by [26]. *α*-weightings are normalised to unity by dividing fluxes through the limiting *V*_max _of each extreme pathway, and are therefore dimensionless values representing the fractional usage of each extreme pathway compared to its maximum capacity.

The sign requirement for an element *w*_*j *_of **w **depends on the nature of elementary mode **E**_*j*_. If all reactions composing **E**_*j *_are reversible, then elementary mode **E**_*j *_is reversible as well and its flux *w*_*j *_may be positive or negative. On the opposite, if **E**_*j *_contains at least one irreversible reaction, then **E**_*j *_is an irreversible elementary mode and *w*_*j *_can only be positive:

*w*_*j *_≥ 0 if **E**_*j *_is irreversible     (4)

It should be noted that condition (4) holds for all *j *if extreme pathways are used instead of elementary modes. Extreme pathways as defined by [10] are always irreversible, since reversible reactions are decomposed into pairs of irreversible reactions.

In general, there are more elementary modes than reactions in a system, and conditions (3) and (4) do not define a unique solution but a continuous convex space of possible solutions. We thus introduced a third condition constraining the system to a unique solution:

∑j=1mwj2
 MathType@MTEF@5@5@+=feaafiart1ev1aaatCvAUfKttLearuWrP9MDH5MBPbIqV92AaeXatLxBI9gBaebbnrfifHhDYfgasaacH8akY=wiFfYdH8Gipec8Eeeu0xXdbba9frFj0=OqFfea0dXdd9vqai=hGuQ8kuc9pgc9s8qqaq=dirpe0xb9q8qiLsFr0=vr0=vr0dc8meaabaqaciaacaGaaeqabaqabeGadaaakeaadaaeWbqaaiabdEha3naaDaaaleaacqWGQbGAaeaacqaIYaGmaaaabaGaemOAaOMaeyypa0JaeGymaedabaGaemyBa0ganiabggHiLdaaaa@378C@ is minimum     (5)

Relations (3), (4) and (5) together define a non-linear optimisation problem, also known as *quadratic programming *problem.

Elementary modes are only unique up to a scaling factor, and the non-linearity of equation (5) makes the solution dependent on the rule decided for scaling elementary modes. It is therefore important to note that numerical values of elementary mode fluxes depend on the scaling strategy. No general rule for scaling elementary modes could be derived from the literature, as this point becomes crucial only with quantitative analysis. Nevertheless, most authors implicitly scale elementary modes to the smallest possible scale allowing all the stoichiometric coefficients they contain to remain integers, and the same rule was therefore applied here.

### Implementation

Our implementation for solving the quadratic programming problem is based on the classical *active set *algorithm, which uses an iterative process. In each iteration, some inequality constraints are set to equality while the remaining constraints are temporarily disregarded, until the correct set of *active constraints *is found. The main steps of our algorithm are described below, while a detailed mathematical demonstration of the active set algorithm can be found in [39].

1a. The algorithm is initialised with a *feasible solution*, that is a vector **w**^(0) ^which fulfils conditions (3) and (4) but is not in general the optimal solution. This step is equivalent to a linear programming problem on its own, and is solved by the simplex method. The obtained solution **w**^(0) ^is used as the first feasible point in step 2, together with an empty active set.

1b. Difficulties in finding a feasible solution may appear if the values of **v **do not strictly verify flux conservation. This may happen because of rounding approximations in numerical computations of flux values, and turned out to occur frequently when steady-state flux distributions were computed by the Gepasi software [33]. Different methods have been developed for balancing inconsistent flux distributions [42]. In our implementation, balancing is achieved through the following steps:

- the null space of the system is computed by a diagonalisation of the matrix of elementary modes,

- the null space is multiplied through the flux vector to verify flux conservation,

- if fluxes are not strictly conserved, a subset of systemically independent fluxes is selected and the remaining fluxes are adjusted to re-establish exact flux conservation.

The previous operations furthermore enable us to reduce the dimension of the quadratic programming problem. In the following steps, only the last subset of systemically independent constraints is kept. This process first guarantees that no degeneracy exists in the constraint set, and second leads to a reduction in computation time that can be worthy for large systems.

2. An equality constraint problem is solved using a feasible point **w**^(*k*) ^and a set of active constraints *A*. This is achieved by shifting the origin to **w**^(*k*) ^and applying the method of Lagrange multipliers. A correction **δ**^(*k*) ^is obtained as a result.

3. If **δ**^(*k*) ^= 0, Lagrange multipliers are computed for the active constraints. If all of them are positive, then **w**^(*k*) ^is the final solution and the algorithm ends. If not, then the constraint corresponding to the lowest Lagrange multiplier is removed from the active set, and the algorithm is repeated from step 2.

4. If **δ**^(*k*) ^is feasible with regard to the constraints not in *A*, then the next iterate is taken as **w**^(*k*+1) ^= **w**^(*k*) ^+ **δ**^(*k*) ^and the algorithm is repeated from step 2. If not, then a line search is made in the direction of **δ**^(*k*) ^until the first inactive constraint becomes active. This constraint is added to the active set *A*, and the algorithm is repeated from step 2.

When the Hessian matrix representing the quadratic optimisation function is positive-definite, as is the case for the function defined by condition (5), then the quadratic problem has a unique global solution. Therefore any solution found in step 3 is the unique global minimum. Successful termination of the active set algorithm has been proved in general [39], excluding the case where degeneracy is present in the constraint set. In this case, the algorithm might cycle by returning to a previously used active set. As the possibility of degeneracy was eliminated in step 1b, that situation should not occur in our implementation, and our algorithm indeed always terminated successfully in our applications.

### Comparison

**Figure 8:**
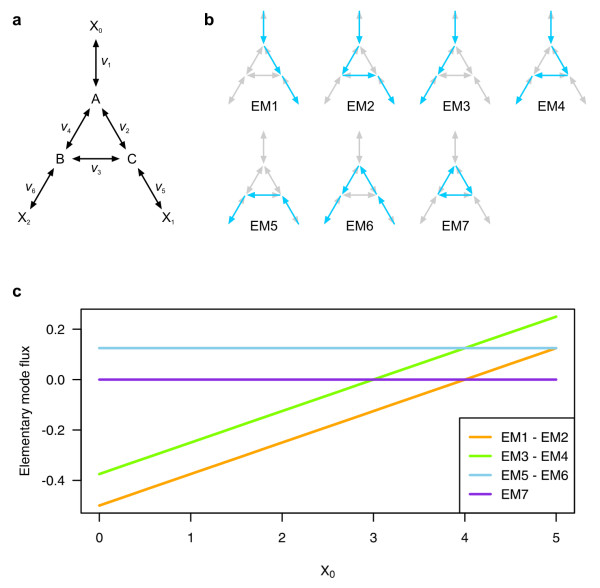
**Theoretical example**. a. Theoretical model presented by [29]. All reactions were modelled as having reversible mass-action kinetics with rate constants equal to one. b. The seven elementary modes of the system, taking *X*_0_, *X*_1 _and *X*_2 _as external metabolites. Since all reactions in the system are reversible, all elementary modes are reversible as well. The direction of blue arrows therefore only refers to a conventional positive direction for elementary mode fluxes; a negative elementary mode flux means that the mode is running in the opposite direction. c. Variations of elementary mode fluxes for concentrations of *X*_0 _ranging from 0 to 5, while *X*_1 _and *X*_2 _have constant concentrations of 4 and 3 respectively.

The approach followed by Poolman *et al. *in [29] for constraining the flux system consisted in inverting the matrix of elementary modes using the Moose-Penrose generalised inverse (as this matrix is generally non-invertible in the classical sense). They showed that this choice eventually leads to the same property as defined by (5), implying that both algorithms should lead to identical results in most cases. For verification we applied our algorithm to the theoretical system presented in [29] and we indeed obtained identical results (Figure 8).

However, the system shown in the previous example contains only reversible fluxes, and thus includes no sign requirement as defined by (4). As a matter of fact, the specificity of the active set algorithm precisely lays in its rigorous treatment of inequality constraints. We therefore cannot exclude that differences between both algorithms would arise when irreversible reactions are present. In that case, when the optimal solution found by Moose-Penrose inversion assigns negative fluxes to some irreversible elementary modes, these modes are assigned zero fluxes in [29], and the inversion is repeated until all assignments to irreversible fluxes are found positive. While this approach may be sufficient to provide the optimal solution in many cases, there is no guarantee that it does so systematically, and one cannot exclude that it might fail in finding a solution if the system enters a loop leading to alternating negative values. More detailed comparisons between both algorithms would need to be conducted using systems containing irreversible fluxes to clarify that point.

## Authors' contributions

JMS initiated the project, carried out the analysis, and wrote the manuscript. MK supervised the project. All authors read and approved the final manuscript.

## Figures and Tables

**Table 1:** List of enzyme and compound abbreviations

Abbreviation	Enzymes, compounds
GLK	glucokinase
PGI	phosphoglucoisomerase
PFK	phosphofructokinase
ALD	fructose-biphosphate aldolase
G3PDH	glycerol-3-phosphate dehydrogenase
GAPDH	glyceraldehyde-3-phosphate dehydrogenase
PGK	3-phosphoglycerate kinase
PGM	phosphoglycerate mutase
ENO	enolase
PYK	pyruvate kinase
PDC	pyruvate decarboxylase
ADH	alcohol dehydrogenase

Glc	glucose
G6P	glucose 6-phosphate
F6P	fructose 6-phosphate
F16P	fructose 1, 6-biphosphate
DHAP	glycerone phosphate
GAP	glyceraldehyde 3-phosphate
BPG	1, 3-biphosphoglycerate
P3G	3-phosphoglycerate
P2G	2-phosphoglycerate
PEP	phosphoenolpyruvate
Pyr	pyruvate
Ace	acetaldehyde
Et	ethanol
Gly	glycerol

**Table 2:** Ranges of allowed variations of kinetic parameters

Enzyme	Parameter	Reference value	Minimum	Maximum
GLK	*V*_max_	226.452	150	100000
	*K*_eq_	3800	1	100000
	*K*_m·Glc_	0.08	0.005	1
	*K*_m·G6P_	20	0.02	1000
	*K*_m·ATP_	0.15	0.0001	0.3
	*K*_m·ADP_	0.23	0.1	1000

PGI	*V*_max_	339.677	30	100000
	*K*_eq_	0.314	0.002	1000
	*K*_m·G6P_	1.4	0.001	100
	*K*_m·F6P_	0.3	0.001	1000

PFK	*V*_max_	182.903	50	100000
	*g*_R_	5.12	0.5	1000
	*L*_0_	0.66	0.1	50000
	*K*_m·ATP_	0.71	0.001	10
	*K*_m·F6P_	0.1	0.001	10
	*K*_AMP_	0.0995	0.0001	10000
	*K*_ATP_	0.65	0.0001	10000
	*K*_F26bP_	0.000682	0.0001	10000
	*K*_F16bP_	0.111	0.0001	10000

ALD	*V*_max_	322.258	100	100000
	*K*_eq_	0.069	0.0005	10
	*K*_m·F16P_	0.3	0.001	50
	*K*_m·GAP_	2	0.0001	10
	*K*_m·DHAP_	2.4	0.001	100
	*K*_m·GAPi_	10	0.02	1000

G3PDH	*V*_max_	70.15	2	300
	*K*_eq_	4300	0.01	100000
	*K*_m·DHAP_	0.4	0.001	500
	*K*_m·NADH_	0.023	0.001	50
	*K*_m·NAD_	0.93	0.001	1000
	*K*_m·Gly_	1	0.0001	1000

GAPDH	*V*_max_	1184.52	200	100000
	*K*_eq_	0.045	0.005	10
	*K*_m·GAP_	0.21	0.001	2
	*K*_m·BPG_	0.0098	0.0005	1000
	*K*_m·NAD_	0.09	0.0001	5
	*K*_m·NADH_	0.06	0.0005	8

PGK	*V*_max_	1306.45	30	100000
	*K*_eq_	3200	100	100000
	*K*_m·BPG_	0.003	0.0001	10
	*K*_m·P3G_	0.53	0.001	30
	*K*_m·ADP_	0.2	0.002	1000
	*K*_m·ATP_	0.3	0.001	30

PGM	*V*_max_	2525.81	50	100000
	*K*_eq_	0.19	0.001	100
	*K*_m·P3G_	1.2	0.001	300
	*K*_m·P2G_	0.08	0.00002	100

ENO	*V*_max_	365.806	200	100000
	*K*_eq_	6.7	0.01	1000
	*K*_m·P2G_	0.04	0.0001	10
	*K*_m·PEP_	0.5	0.00003	100

PYK	*V*_max_	1088.71	300	100000
	*K*_eq_	6500	0.1	9000
	*K*_m·PEP_	0.14	0.01	9
	*K*_m·Pyr_	21	0.1	1000
	*K*_m·ADP_	0.53	0.01	4
	*K*_m·ATP_	1.5	0.3	100

PDC	*V*_max_	174.194	100	100000
	*K*_m·Pyr_	4.33	0.001	50

ADH	*V*_max_	810	200	100000
	*K*_eq_	6.9·10^-5^	10^-8^	0.0005
	*K*_m·Ace_	1.11	0.01	1000
	*K*_m·Et_	17	0.001	1000
	*K*_m·NADH_	0.11	0.001	5
	*K*_m·NADP_	0.17	0.001	30

**Table 3:** Standard deviations of elementary mode fluxes under variation of single kinetic parameters, and elasticity coefficients of parameters at the experimental steady state

Enzyme	Parameter	EM1	EM2	EM3	EM4	EM5	EM6	EM7	EM8	Elasticity
GLK	*V*_max_	0	0	0	0	0.12	0	0.58	4.01	1.0
	*K*_eq_	0	0	0	0.21	0.48	0	1.85	8.94	0.0014
	*K*_m·Glc_	0	0	0	0.23	0.58	0	2.30	12.02	-0.456
	*K*_m·G6P_	0	0	0	0.21	0.52	0	1.99	9.80	0.015
	*K*_m·ATP_	0	0	0	0	0.05	0	0.27	1.91	-0.283
	*K*_m·ADP_	0	0	0	0	0.06	0	0.31	2.15	0.241

PGI	*V*_max_	0	0	0	0.27	0.62	0	2.30	10.12	1.0
	*K*_eq_	0	0	0	0	0.05	0	0.27	1.90	0.533
	*K*_m·G6P_	0	0	0	0	0.04	0	0.21	1.48	-0.651
	*K*_m·F6P_	0	0	0	0	0.04	0	0.01	0.02	0.178

PFK	*V*_max_	0	0	0	0.09	0.29	0	1.17	6.27	1.0
	*g*_R_	0	0	0	0	0.01	0	0.05	0.32	0.937
	*L*_0_	0	0	0	0	0.10	0	0.50	3.53	-0.460
	*K*_m·ATP_	0	0	0	0	0	0	0.01	0.06	-0.086
	*K*_m·F6P_	0	0	0	0	0.05	0	0.26	1.80	-0.934
	*K*_AMP_	0	0	0	0	0	0	0.02	0.15	-0.504
	*K*_ATP_	0	0	0	0	0	0	0.01	0.06	0.187
	*K*_F2bP_	0	0	0	0	0.01	0	0.03	0.18	-0.626
	*K*_F16bP_	0	0	0	0	0	0	0.02	0.16	0.401

ALD	*V*_max_	0	0	0	0	0	0	0	0.03	1.0
	*K*_eq_	0	0	0	0	0	0	0.02	0.13	1.499
	*K*_m·F16P_	0	0	0	0	0	0	0.01	0.09	-0.398
	*K*_m·GAP_	0	0	0	0	0	0	0.01	0.10	0.0067
	*K*_m·DHAP_	0	0	0	0	0	0	0.02	0.11	0.094
	*K*_m·GAPi_	0	0	0	0	0	0	0	0.01	0.0020

G3PDH	*V*_max_	0	0	0.87	1.96	1.24	0	7.65	13.74	1.0
	*K*_eq_	0	0	0	0	1.40	0	6.98	8.90	0.0016
	*K*_m·DHAP_	0	0	0	0.38	1.60	0	8.77	11.88	-0.382
	*K*_m·NADH_	0	0	0	1.87	1.28	0	8.50	13.81	-0.579
	*K*_m·NAD_	0	0	0	0.39	1.57	0	8.65	11.80	0.362
	*K*_m·Gly_	0	0	0	0	1.39	0	6.94	8.88	0.050

GAPDH	*V*_max_	0	0	0	0.17	1.35	0	6.98	9.49	1.0
	*K*_eq_	0	0	0	0.10	1.25	0	6.37	8.66	0.880
	*K*_m·GAP_	0	0	0	0.12	1.48	0	7.58	10.23	-0.919
	*K*_m·BPG_	0	0	0	0	0.17	0	0.83	1.16	0.082
	*K*_m·NAD_	0	0	0	0.17	0.31	0	1.81	2.79	-0.145
	*K*_m·NADH_	0	0	0	0.15	0.30	0	1.67	2.53	0.092

PGK	*V*_max_	0	0	0	0.02	0.23	0	1.17	1.70	1.0
	*K*_eq_	0	0	0	0.02	0.28	0	1.44	2.06	2.909
	*K*_m·BPG_	0	0	0	0	0.02	0	0.11	0.16	0.062
	*K*_m·P3G_	0	0	0	0.17	0.16	0	1.13	1.87	-0.623
	*K*_m·ADP_	0	0	0	0.06	0.18	0	1.07	1.63	0.408
	*K*_m·ATP_	0	0	0	0.15	0.18	0	1.17	1.90	-0.471

PGM	*V*_max_	0	0	0.56	0.77	0.70	0	2.35	9.51	1.0
	*K*_eq_	0	0	0	0.06	0.29	0	1.56	2.28	1.959
	*K*_m·P3G_	0	0	0	0	0.25	0	1.27	1.81	-0.840
	*K*_m·P2G_	0	0	0	0.18	0.27	0	1.63	2.57	0.302

ENO	*V*_max_	0	0	0	0	0.03	0	0.14	0.19	1.0
	*K*_eq_	0	0	0	0.10	0.27	0	1.48	2.22	0.325
	*K*_m·P2G_	0	0	0	0.06	0.28	0	1.50	2.19	-0.506
	*K*_m·PEP_	0	0	0	0.25	0.27	0	1.75	2.87	0.065

PYK	*V*_max_	0	0	0	0	0.08	0	0.40	0.56	1.0
	*K*_eq_	0	0	0	0.23	0.28	0	1.76	2.85	0.036
	*K*_m·PEP_	0	0	0	0	0.16	0	0.82	1.15	-0.728
	*K*_m·Pyr_	0	0	0	0	0.15	0	0.73	1.02	0.210
	*K*_m·ADP_	0	0	0	0	0.17	0	0.84	1.19	-0.523
	*K*_m·ATP_	0	0	0	0	0.02	0	0.08	0.13	0.327

PDC	*V*_max_	0	0	0	0	0	0	0.02	0.02	1.0
	*K*_m·Pyr_	0	0	0	0	0.02	0	0.09	0.13	-0.411

ADH	*V*_max_	0	0	0	0.05	0.37	0	1.94	2.70	1.0
	*K*_eq_	0	0	0	1.26	1.33	0	7.80	11.76	-3.393
	*K*_m·Ace_	0	0	0.76	1.53	0.61	0	1.61	5.80	0.139
	*K*_m·Et_	0	0	1.00	1.77	0.79	0	1.72	7.84	-0.457
	*K*_m·NADH_	0	0	0	0.37	0.23	0	1.54	2.62	-0.113
	*K*_m·NADP_	0	0	0.97	1.84	0.83	0	1.74	7.24	-0.102

**Table 4:** Standard deviations of elementary mode fluxes under variation of all kinetic parameters of each enzyme

Enzymes	EM1	EM2	EM3	EM4	EM5	EM6	EM7	EM8
GLK	0	0	0	0.04	0.22	0	1.02	6.60
PGI	0	0	0	0.16	0.38	0	1.46	6.99
PFK	0	0	0	0.01	0.12	0	0.61	4.09
ALD	0	0	0	0	0	0	0.02	0.13
G3PDH	0	0	0.27	0.49	0.83	0	4.45	23.07
GAPDH	0	0	0	0.06	1.40	0	7.06	9.35
PGK	0	0	0.18	0.27	0.34	0	1.59	3.61
PGM	0	0	0.26	0.38	0.44	0	2.05	4.55
ENO	0	0	0	0.13	0.31	0	1.66	2.48
PYK	0	0	0	0.23	0.27	0	1.69	2.73
PDC	0	0	0	0.05	0.06	0	0.40	0.66
ADH	0	0	0.70	1.30	1.34	0	7.78	13.81

All enzymes	0	0	0.47	0.64	0.61	0	3.41	20.58

**Table 5:** Standard deviations of elementary mode fluxes under variation of concentrations of external compounds

Concentrations	EM1	EM2	EM3	EM4	EM5	EM6	EM7	EM8
Glucose	0	0	0	0.05	0.17	0	0.77	4.66
Ethanol	0	0	0	1.82	0.99	0	5.72	10.30

All concentrations	0	0	0.40	2.22	1.23	0	2.35	6.90

**Table 6:** Control coefficients of elementary mode fluxes by individual steps at the experimental steady state. The bottom part of the table refers to the following processes: GLT, transport of glucose across the cell membrane; Glyco, glycogen branch; Treha, trehalose branch; Succ, succinate branch; AK, equilibrium constraint on adenylate kinase due to the conservation of adenine nucleotides.

Enzymes, processes	EM1	EM2	EM3	EM4	EM5	EM6	EM7	EM8
GLK	0	0	N/A	N/A	0.090	N/A	0.090	0.208
PGI	0	0	N/A	N/A	0.0013	N/A	0.0010	0.0030
PFK	0	0	N/A	N/A	0.0010	N/A	0.0009	0.0023
ALD	0	0	N/A	N/A	0.0002	N/A	0.0005	0.0004
G3PDH	0	0	N/A	N/A	0.563	N/A	0.562	-0.249
GAPDH	0	0	N/A	N/A	-0.246	N/A	-0.246	0.111
PGK	0	0	N/A	N/A	-0.0068	N/A	-0.0057	0.0026
PGM	0	0	N/A	N/A	-0.0056	N/A	-0.0049	0.0023
ENO	0	0	N/A	N/A	-0.015	N/A	-0.015	0.0067
PYK	0	0	N/A	N/A	-0.0076	N/A	-0.0072	0.0033
PDC	0	0	N/A	N/A	-0.0045	N/A	-0.0042	0.0019
ADH	0	0	N/A	N/A	-0.108	N/A	-0.109	0.049

GLT	0	0	N/A	N/A	0.573	N/A	0.574	1.316
Glyco	0	1	N/A	N/A	-0.056	N/A	-0.056	-0.115
Treha	1	0	N/A	N/A	-0.043	N/A	-0.043	-0.090
Succ	0	0	N/A	N/A	0.346	N/A	0.346	-0.153
AK	0	0	N/A	N/A	-0.093	N/A	-0.093	-0.095

Summation	1	1	N/A	N/A	0.989	N/A	0.990	1.004
